# Atypical Adult Still’s Disease Complicated by Hemophagocytic Syndrome in an Older Patient: A Case Report

**DOI:** 10.7759/cureus.46922

**Published:** 2023-10-12

**Authors:** Minami Kakehi, Shiho Amano, Chiaki Sano, Ryuichi Ohta

**Affiliations:** 1 Family Medicine, Shimane University Medical School, Izumo, JPN; 2 Community Care, Unnan City Hospital, Unnan, JPN; 3 Community Medicine, Shimane University Faculty of Medicine, Izumo, JPN; 4 Communiy Care, Unnan City Hospital, Unnan, JPN

**Keywords:** invasive intervention, sepsis, fever of unknown origin, interleukin-6, pancytopenia, tocilizumab, atypical adult still's disease, hemophagocytic syndrome, macrophage activation syndrome, hyperferritinemia

## Abstract

Hyperferritinemia can occur in various diseases, making the differential diagnoses diverse and often fatal. The macrophage-activated syndrome (MAS) is a differential diagnosis of hyperferritinemia in which systemic macrophages are activated and cause various symptoms. Many cases are complicated by hemophagocytic syndrome, causing pancytopenia, which can be fatal. Furthermore, it is challenging to diagnose hyperferritinemia in elderly patients, and the disease may develop into a fever of unknown origin. We report the case of a 93-year-old man with aspiration pneumonia, followed by intermittent prolonged fever complicated by abnormal hyperferritinemia and leukopenia. Based on his general condition, he was diagnosed with atypical adult Still's disease and treated with steroid pulses and tocilizumab, temporarily relieving his symptoms. However, the patient eventually developed sepsis and could not be saved. Diagnosis of hyperferritinemia in the elderly population is complex and requires immediate attention. However, invasive intervention may lead to the deterioration of an elderly patient's condition. In the context of medical care for the elderly at a community hospital, it is necessary to provide comprehensive care for those in critical condition, considering the degree of invasiveness of examinations and procedures.

## Introduction

Hyperferritinemia may be accompanied by a fever found at close examination, and the differential diagnoses in such cases are diverse [1]. In the diagnosis of hyperferritinemia, the differential diagnosis differs according to the degree of its elevation [2]. In particular, the differential diagnosis is significantly narrowed when abnormally high ferritin levels of 3,000 ng/mL are observed [3]. One such condition with abnormally high ferritin levels is macrophage-activated syndrome (MAS) [4]. Abnormal activation of macrophages leads to phagocytosis of blood cells in the bone marrow, leading to elevated ferritin levels [1]. Immunological abnormalities often cause this, and it has been suggested that inflammatory cytokines such as interleukin-6 (IL-6) may affect the elevated ferritin level, and their elevation may contribute to diagnosis and treatment [5].

Abnormally high ferritin levels were observed in an older male patient with progressive generalized fatigue and fever. Sepsis was initially suspected, but the patient developed a prolonged fever. During the disease, pancytopenia was observed, IL-6 levels were elevated, and no prominent malignant findings were observed on close examination; therefore, a diagnosis of MAS due to adult Still's disease was made. Steroid pulse therapy and tocilizumab were administered, the inflammation subsided, and the patient's general condition temporarily stabilized. In this case, we discuss the importance of appropriate systemic management and follow-up for elderly patients with abnormally high ferritin levels and the necessity of smooth, multidisciplinary treatment according to the patient's condition.

## Case presentation

A 93-year-old man living at home without needing nursing care visited a rural community hospital for several weeks because of a prolonged fever. He had a fever of 37.8 °C, coughing, and nasal discharge during the three weeks of his visits; therefore, he visited a primary care doctor who prescribed acetaminophen of 1,500 mg per day, and his cough and nasal discharge became mild with symptomatic treatment. The patient continued to have a fever of 37.0-37.8 °C and was referred to our hospital. He had a history of cerebral infarction and had been taking aspirin (100 mg).

Vital signs at admission were a temperature of 38.1 °C; pulse, 106/min; blood pressure, 137/86 mmHg; respiratory rate, 30/min; and oxygen saturation, 95% (room air). Physical examination revealed no abnormalities in the head and neck regions, and late crackles were heard at the base of both lungs without the usage of respiratory accessory muscles. No prominent abdominal or extremity findings were observed. The laboratory data revealed high C-reactive protein and ferritin and suppressing serum albumin levels, showing high inflammatory condition (Table 1).

**Table 1:** Initial laboratory results of the patient CK, creatine kinase; CRP, C-reactive protein; eGFR, estimated glomerular filtration rate; SARS-CoV-2, severe acute respiratory syndrome coronavirus 2.

Parameter	Level	Reference
White blood cells (× 10^3^/μL)	5.4	3.5–9.1
Neutrophils (%)	69.1	44.0–72.0
Lymphocytes (%)	9.3	18.0–59.0
Monocytes (%)	21.1	0.0–12.0
Eosinophils (%)	0.2	0.0–10.0
Basophils (%)	0.3	0.0–3.0
Red blood cells (× 10^6^/μL)	4.30	3.76–5.50
Hemoglobin (g/dL)	13.3	11.3–15.2
Hematocrit (%)	38.5	33.4–44.9
Mean corpuscular volume (fL)	89.4	79.0–100.0
Platelets (× 10^4^/μL)	18.8	13.0–36.9
Total protein (g/dL)	5.9	6.5–8.3
Albumin (g/dL)	2.9	3.8–5.3
Total bilirubin (mg/dL)	0.8	0.2–1.2
Aspartate aminotransferase (IU/L)	34	8–38
Alanine aminotransferase (IU/L)	24	4–43
Alkaline phosphatase (U/L)	172	106–322
γ-Glutamyl transpeptidase (IU/L)	69	<48
Lactate dehydrogenase (mg/dL)	249	121–245
Blood urea nitrogen (mg/dL)	11.0	8–20
Creatinine (mg/dL)	0.54	0.40–1.10
eGFR (mL/min/1.73 m^2^)	90	>60.0
Serum Na (mEq/L)	128	135–150
Serum K (mEq/L)	3.6	3.5–5.3
Serum Cl (mEq/L)	92	98–110
Serum Ca (mg/dL)	8.1	8.8–10.2
Serum P (mg/dL)	2.4	2.7–4.6
Serum Mg (mg/dL)	1.6	1.8–2.3
CK (U/L)	23	56–244
CRP (mg/dL)	12.04	<0.30
Ferritin (ng/mL)	2082.1	14.4-303.7
SARS-CoV-2 antigen	Negative	Negative
Urine analysis		
Leukocyte	Negative	Negative
Nitrite	Negative	Negative
Protein	Negative	Negative
Glucose	Negative	Negative
Urobilinogen	normal	normal
Bilirubin	Negative	Negative
Ketones	Negative	Negative
Blood	Negative	Negative
pH	7.0	NA
Specific gravity	1.024	NA

Chest radiography and computed tomography (CT) revealed no infiltrates. Sputum Gram staining revealed white blood cells, gram-positive cocci, and negative rods. Treatment with ampicillin/sulbactam (6 g/day) was started for bronchial pneumonia. The patient continued to have a persistent fever of around 38 ºC. Contrast-enhanced CT scans of the chest and pelvic region revealed no apparent abscesses or mass lesions. Sputum Gram staining did not reveal any organisms. Quantitative mycoplasma, influenza, and coronavirus antigen tests yielded negative results. The fever was considered drug-induced on the fifth day of admission, and antimicrobial therapy was terminated. His fever resided within five days of the withdrawal of the antibiotic.

**Figure 1:**
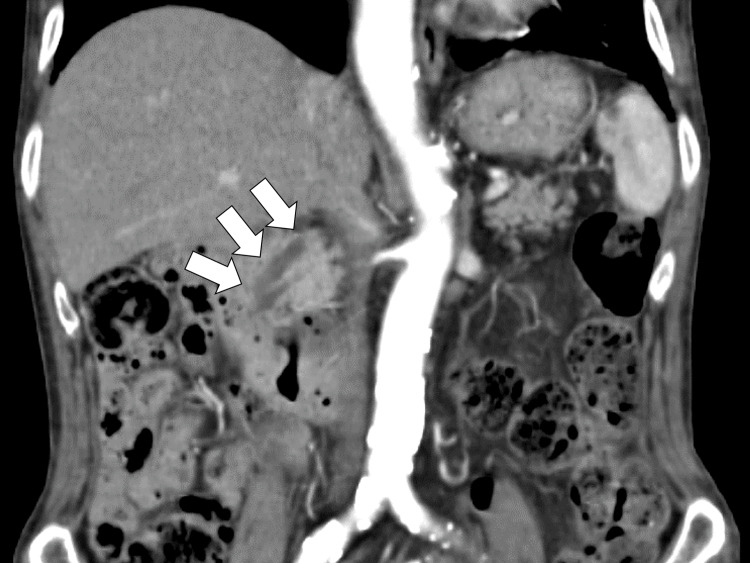
Endoscopic retrograde cholangiopancreatography revealing mild dilatation of the common bile duct without stones (White arrows)

While he was rehabilitated for the preparation of discharge, on day 25 after admission, the patient presented a fever of around 38 °C and general malaise. He continued to be relatively bradycardic, and contrast-enhanced CT of the chest and pelvis showed mild dilatation of the common bile duct with small cholangial stones and no significant lymphadenopathy. The patient had high ferritin levels (7,812.7 ng/dL) and soluble Interleuken-2 receptor (9,031 U/mL (reference, 157-474 U/mL)). Endoscopic retrograde cholangiopancreatography (ERCP) revealed mild dilatation of the common bile duct without stones (Figure 1).

The culture of bile did not show any infection. The dermatology department performed a random skin biopsy upon suspicion of intravascular lymphoma. However, the biopsy revealed only an infiltrate of normal lymphocytes in the skin. The patient developed cachexia secondary to inflammation. Eventually, he was diagnosed with adult-onset Still's disease and was treated orally with prednisolone (60 mg/day). The fever resolved promptly, and the activities of daily living improved.

On day 41 of admission, the patient became febrile when the prednisolone dose was reduced to 40 mg/day. Fever with elevated hepatobiliary enzyme and ferritin levels appeared rapidly, and pancytopenia developed. Based on the patient’s history, the possibility of hemophagocytic syndrome (HLH) due to adult Still's disease was considered. Tocilizumab infusion (200 mg) was initiated. Inflammatory conditions and ferritin levels decreased, and the platelet count increased.

On day 60 of admission, the patient developed tachycardia and tenderness in the abdomen, which were suspected to be peritonitis due to bacterial translocation by intestinal bacteria. Meropenem (3g/day) was started. Unfortunately, the patient's symptoms continued progressing, and he died on day 66 after admission.

## Discussion

In this case, pneumonia was initially thought to be due to upper respiratory tract symptoms; however, fever and ferritin elevation continued, and the patient was eventually treated for MAS due to adult-onset Still's disease after ruling out obvious malignancy. It is essential to recognize that an abnormally high ferritin level in an elderly patient should exclude fatal diseases. Additionally, MAS is complicated by adult-onset Still's disease in elderly patients and can be fatal.

It is essential to establish an appropriate differential diagnosis and rule out urgent diseases when ferritin levels are elevated in the elderly population. In this case, a final diagnosis of MAS due to adult Still's disease was made; however, sepsis and disseminated intravascular coagulation were ruled out. Concurrent diseases of the patient should be closely evaluated for bacteremia and malignancy [6]. In the present case, there was no obvious mass lesion, and a random skin biopsy showed no prominent malignant findings, with only normal lymphocyte infiltration. Additionally, differentiation based on ferritin levels is essential. In previous literature, adult Still’s disease, complicated by MAS, is the most common condition in which ferritin is 3,000 ng/mL or higher [7]. In addition, a typical skin rash is not often observed in Asian and elderly patients, which may make diagnosis difficult [8,9]. Similar cases may have increased with the advent of hyper-aged societies. In cases of unexplained fever, ferritin measurement and prompt treatment, based on the measurement results, are necessary.

A smooth diagnosis by exclusion and risk-based empiric therapy is vital in treating progressive diseases with abnormally high ferritin levels. In the present case, it was difficult to exclude sepsis because the patient had a slowly progressive fever, general malaise, was elderly, and immunosuppressed [9]. The patient's general condition suggested bacteremia, which could have been caused by bacterial translocation from the kidneys and intestinal tract. In immunosuppressed patients, it is necessary to consider infection by commensal bacteria in the urinary and intestinal tracts; in this case, extended-spectrum beta-lactamase-producing bacteria were suspected, and treatment was initiated. However, the patient had a persistent fever with a common bile duct stone on CT, leading us to consider the possibility of a hepatobiliary infection. However, further deterioration of the patient's general condition was observed after the ERCP. Invasive procedures in elderly patients may exacerbate MAS [10]. In our patient, fever persisted after ERCP and abnormally high ferritin levels and progression of pancytopenia were observed. At this stage, the possibility of MAS due to adult Still's disease increased, and a steroid pulse was administered. Considering the possibility of further inflammation, tocilizumab was administered. Subsequently, his consciousness improved to the point where he could interact.

In elderly patients with fever and abnormally high ferritin levels, it is essential to initiate antimicrobial therapy while ruling out sepsis [11]. It is also necessary to monitor the patient's progress while considering immunosuppression and avoiding excessively invasive treatment [12]. Based on the progression of symptoms, it was considered safe to consider subacute inflammatory diseases, including adult Still's disease, and administer additional treatments. In community hospitals, it is essential to treat the symptoms of elderly patients by treating the entire body as a system [13]. Patient safety should be considered while administering treatment. Thus, immunity should be treated as an essential element for interpreting the system and responding to fever in the elderly.

## Conclusions

In cases of persistent fever in the elderly, close examination for hyperferritinemia can explain the diagnosis. It is crucial to proceed with a close examination and treatment while considering the immunosuppressive status of the elderly population. In a hyper-aged society, medical care needs to understand the general condition of the elderly systematically through prompt empirical treatment.
